# TGF-β Inhibition Restores Terminal Osteoblast Differentiation to Suppress Myeloma Growth

**DOI:** 10.1371/journal.pone.0009870

**Published:** 2010-03-25

**Authors:** Kyoko Takeuchi, Masahiro Abe, Masahiro Hiasa, Asuka Oda, Hiroe Amou, Shinsuke Kido, Takeshi Harada, Osamu Tanaka, Hirokazu Miki, Shingen Nakamura, Ayako Nakano, Kumiko Kagawa, Kenichiro Yata, Shuji Ozaki, Toshio Matsumoto

**Affiliations:** 1 Department of Medicine and Bioregulatory Sciences, University of Tokushima Graduate School of Medicine, Tokushima, Japan; 2 Department of Biomaterials and Bioengineering, University of Tokushima Graduate School of Oral Sciences, Tokushima, Japan; 3 Division of Transfusion Medicine, Tokushima University Hospital, Tokushima, Japan; Medical College of Georgia, United States of America

## Abstract

**Background:**

Multiple myeloma (MM) expands almost exclusively in the bone marrow and generates devastating bone lesions, in which bone formation is impaired and osteoclastic bone resorption is enhanced. TGF-β, a potent inhibitor of terminal osteoblast (OB) differentiation, is abundantly deposited in the bone matrix, and released and activated by the enhanced bone resorption in MM. The present study was therefore undertaken to clarify the role of TGF-β and its inhibition in bone formation and tumor growth in MM.

**Methodology/Principal Findings:**

TGF-β suppressed OB differentiation from bone marrow stromal cells and MC3T3-E1 preosteoblastic cells, and also inhibited adipogenesis from C3H10T1/2 immature mesenchymal cells, suggesting differentiation arrest by TGF-β. Inhibitors for a TGF-β type I receptor kinase, SB431542 and Ki26894, potently enhanced OB differentiation from bone marrow stromal cells as well as MC3T3-E1 cells. The TGF-β inhibition was able to restore OB differentiation suppressed by MM cell conditioned medium as well as bone marrow plasma from MM patients. Interestingly, TGF-β inhibition expedited OB differentiation in parallel with suppression of MM cell growth. The anti-MM activity was elaborated exclusively by terminally differentiated OBs, which potentiated the cytotoxic effects of melphalan and dexamethasone on MM cells. Furthermore, TGF-β inhibition was able to suppress MM cell growth within the bone marrow while preventing bone destruction in MM-bearing animal models.

**Conclusions/Significance:**

The present study demonstrates that TGF-β inhibition releases stromal cells from their differentiation arrest by MM and facilitates the formation of terminally differentiated OBs, and that terminally differentiated OBs inhibit MM cell growth and survival and enhance the susceptibility of MM cells to anti-MM agents to overcome the drug resistance mediated by stromal cells. Therefore, TGF-β appears to be an important therapeutic target in MM bone lesions.

## Introduction

Multiple myeloma (MM) develops and expands almost exclusively in the bone marrow and generates devastating bone lesions. In typical destructive bone lesions in patients with MM, bone formation is impaired along with an enhancement of osteoclastic bone resorption. We and others have demonstrated that MM cells enhance osteoclastogenesis by MIP-1 and RANK ligand,[1], [2], [3], [4]while suppressing osteoblast (OB) differentiation from their precursors, stromal cells, via the secretion of soluble Wnt antagonists from MM cells,[5], [6], [7] stromal cells and OBs.[8], [9] Thus induced osteoclasts (OCs) as well as stromal cells with defective OB differentiation in turn enhance MM cell growth and survival.[10], [11] Furthermore, OCs stimulate angiogenesis in concert with MM cells.[12] These MM cell-induced cell types in MM bone lesions, namely OCs, vascular endothelial cells and stromal cells, create a microenvironment suitable for MM cell growth and survival, which can be called as a “MM niche”. [13] Because such a skewed cellular microenvironment protects MM cells from apoptosis induced by chemotherapeutic agents as well as immunotherapy, there is a need to target and disrupt the MM niche to improve the efficacy of present therapeutic modalities against MM progression as well as MM bone disease.

Bone marrow stromal cells with defective OB differentiation are a major component of the MM niche, which produce various growth and anti-apoptotic factors for MM cells including IL-6, IGF-1, SDF-1α and VEGF while expressing RANK ligand to stimulate osteoclastogenesis. Importantly, the adhesion of MM cells to stromal cells as well as their extracellular matrices (ECM) confers cell adhesion-mediated drug resistance (CAM-DR) in MM cells.[14], [15], [16] Therefore, there is a possibility that induction of OB differentiation in stromal cells not only prevents bone loss and resumes bone formation in MM bone lesions, but also may perturb MM growth enhanced by stromal cells.

TGF-β, a potent inhibitor of terminal OB differentiation and mineralization,[17], [18], [19] is produced by OBs and osteocytes, and abundantly deposited in bone matrices in a latent form.[20] It is released from bone matrices through bone resorption[21] and activated by acids and matrix metalloproteinases secreted from OCs.[22], [23], [24] Because osteoclastic bone resorption is enhanced in MM, TGF-β appears to be abundant and active in MM bone lesions, and may play an important role in bone formation impaired by MM. Therefore, the present study was undertaken to explore whether an inhibition of TGF-β enhances OB differentiation suppressed by MM, and whether an enhancement of OB differentiation affects MM cell growth and survival. We demonstrate herein that a blockade of TGF-β actions releases stromal cells from their differentiation arrest by MM, and that terminally differentiated OBs inhibit MM cell growth and survival and potentiate responsiveness to anti-MM agents. These results suggest that suppression of OB differentiation by MM not only accelerates bone loss but also creates a MM niche to enhance MM growth and survival. Thus, an induction of OB differentiation through TGF-β inhibition may provide a novel approach to ameliorate both bone destruction and tumor progression in MM.

## Results

### TGF-β suppresses and TGF-β inhibitors enhance OB differentiation

**Figure 1:**
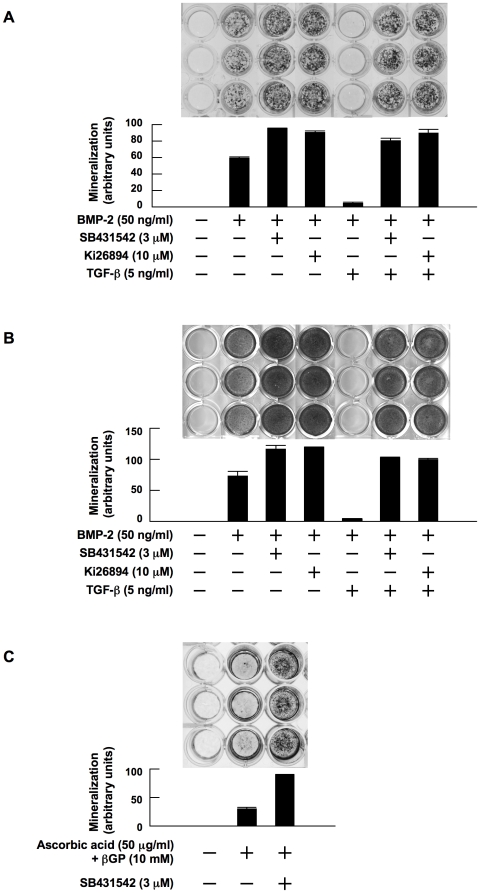
TGF-β suppresses and TGF-β inhibitors enhance OB differentiation. MC3T3-E1 cells (**A**) and primary bone marrow stromal cells (**B**) were cultured for 14 and 28 days, respectively, in 24-well culture plates in α-MEM containing 10% FBS supplemented with β-glycerophosphate and ascorbic acid (osteogenic medium). rhBMP-2, SB431542, Ki26894 and rhTGF-β were added at 50 ng/mL, 3 µM, 10 µM and 5 ng/mL to the indicated wells, respectively. After culturing, mineralized nodules were visualized by von Kossa staining. **C.** MC3T3-E1 cells were cultured for 21 days in osteogenic media in the absence of BMP-2. SB431542 was added at 3 µM to the indicated wells. Mineralized nodules were visualized by von Kossa staining. The data with mineralized nodule formation were quantified by densitometric analyses.

Because OB differentiation from stromal cells is impaired in MM bone lesions, and because TGF-β is activated through enhanced bone resorption, we first examined the effect of TGF-β and its inhibition on OB differentiation. Addition of BMP-2 induced OB differentiation with the formation of mineralized nodules by MC3T3-E1 preosteoblastic cells (Figure 1A) and primary bone marrow stromal cells from a patient with MM (Figure 1B). Interestingly, treatment with inhibitors of the TGF-β type I receptor kinase ALK5, SB431542 and Ki26894, to inhibit endogenous TGF-β increased the formation of mineralized nodules. To simulate TGF-β-rich MM bone lesions, we added TGF-β, and examined OB differentiation in the presence of TGF-β. Addition of TGF-β almost completely suppressed the BMP-2-induced formation of mineralized nodules. However, treatment with SB431542 or Ki26894 restored the mineralization on addition of TGF-β. In addition, SB431542 alone facilitated osteogenesis to achieve mineralized nodule formation by MC3T3-E1 cells even in the absence of BMP-2 (Figure 1C), which may be due to the suppression of endogenous TGF-β produced by MC3T3-E1 cells. Thus, TGF-β suppresses and its inhibition enhances OB differentiation.

### TGF-β suppresses adipogenic differentiation as well as OB differentiation from immature mesenchymal cells

**Figure 2:**
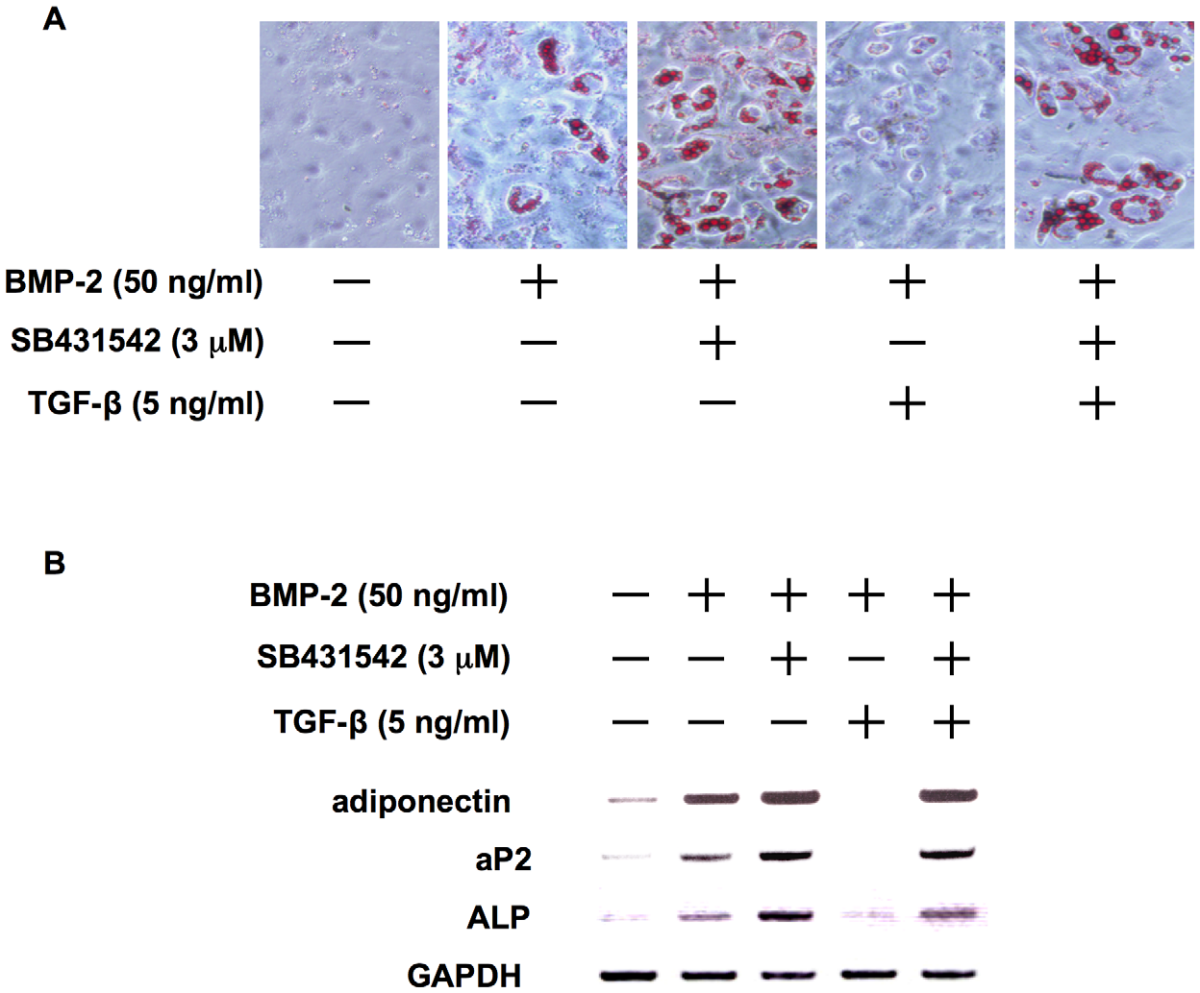
TGF-β suppresses adipogenic differentiation as well as OB differentiation from immature mesenchymal cells. **A.** C3H10T1/2 immature mesenchymal cells were cultured in osteogenic medium for 7 days. rhBMP-2, SB431542 and rhTGF-β were added at 50 ng/mL, 3 µM and 5 ng/mL to the indicated wells, respectively. After culturing for 7days, the cells were stained with oil red O. Representative images of oil red O staining are shown. **B.** C3H10T1/2 cells were harvested after culturing for 2 days. rhBMP-2, SB431542 and rhTGF-β were added at 50 ng/mL, 3 µM and 5 ng/mL to the indicated wells, respectively. mRNA expression of markers for adipogenic and osteoblastic differentation were analyzed by RT-PCR. The primers used were as follows: Mouse adioponectin sense 5′-AGGGTGAGACAGGAGATGTTGGAA-3′ and antisense 5′-CAGAGGCCTGGTCCACATTCTTTT-3′. Mouse aP2 sense 5′-TCTCACCTGGAAGACAGCTCCTCCTCG-3′ and antisense 5′-TTCCATCCAGGCCTCTTCCTTTGGCTC-3′. Mouse ALP sense 5′-CACTCAGGGCAATGAGGTCACATC-3′ and antisense 5′-TTCAGTGCGGTTCCAGACATAGTG-3′.

In addition to OB differentiation, BMP-2 can induce the differentiation of mesenchymal stem cells or pluripotent C3H10T1/2 immature mesenchymal cells into adipocytes .[25], [26] When C3H10T1/2 cells were cultured in an osteogenic medium with BMP-2, significant numbers of oil red O-positive adipocytes appeared (Figure 2A). Addition of SB431542 to suppress endogenous TGF-β actions enhanced adipogenic differentiation by BMP-2. TGF-β almost completely suppressed adipogenic differentiation, which resumed with the addition of SB431542. Also, the mRNA expression of adipogenic differentiation markers including adioponectin and aP2 was induced along with the expression of ALP, a marker for OBs, by BMP-2, which was further up-regulated by TGF-β inhibition (Figure 2B). TGF-β suppressed the expression of both the adipogenic and OB differentiation markers, which was reversed by TGF-β inhibition. Thus, TGF-β suppresses adipogenic as well as osteoblastic differentiation in C3H10T1/2 cells, suggesting immaturity of undifferentiated mesenchymal cells or stromal cells retained by TGF-β.

### TGF-β inhibition restores and enhances OB differentiation suppressed by MM

**Figure 3:**
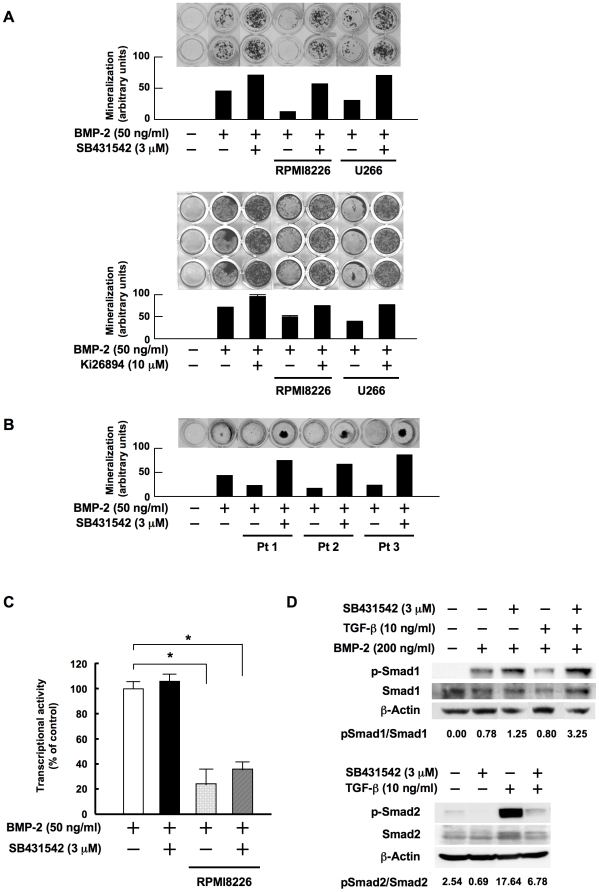
TGF-β inhibition restores OB differentiation and potentiates Smad1 phosphorylation by BMP-2. **A.** MC3T3-E1 cells were cultured for 14 days in osteogenic media in the absence or presence of conditioned media from RPMI8226 and U266 cells at 20%. SB431542 and Ki26894 were added at 3 and 10 µM to the indicated wells, respectively. **B.** MC3T3-E1 cells were cultured for 14 days in osteogenic media in the absence or presence of bone marrow plasma from patients with MM at 5%. SB431542 was added at 3 µM to the indicated wells. **C.** A chimeric firefly luciferase reporter plasmid (Topflash) and a renilla luciferase reporter plasmid (pRL-TK) were cotransfected into MC3T3-E1 cells as described in Materials and methods. The MC3T3-E1 cells were cultured in osteogenic media with rhBMP-2 at 50 ng/ml in the absence or presence of conditioned medium from RPMI8226 cells at 10%. SB431542 was added at 3 µM to the indicated wells. After 12 hours of incubation luiferase reporter activity was measured as renilla luiferase activity. Data were expressed as means +/− SD for six independent experiments. **D.** MC3T3-E1 cells were cultured in α-MEM with 1% FBS for 24 hours in 10-cm dishes at 60% confluence. SB431542 (3 µM), rhTGF-β (10 ng/mL), and the two in combination were added to the indicated dishes and the cells were cultured for another 48 hours, after which rhBMP-2 was added at 200 ng/ml (upper). SB431542 (3 µM), rhTGF-β (10 ng/mL), and the two in combination were added to the indicated dishes (lower). Following incubation for 30 minutes, cell lysates were collected. The protein levels of phosphorylated Smad1, Smad1, phosphorylated Smad2 and Smad2 were estimated by Western blotting. β-actin was used as a protein loading control.

Because MM cells produce soluble Wnt antagonists and suppress OB differentiation,[5], [6], [7] we next examined whether TGF-β inhibition can restore OB differentiation suppressed by MM cells. Conditioned media from MM cell lines suppressed the development of mineralized nodules stimulated by BMP-2 (Figure 3A). Addition of SB431542 or Ki26894 restored the modulation in the presence of MM cell conditioned media to a level more than BMP-2 alone, suggesting that TGF-β inhibition antagonizes the suppression of OB differentiation by MM cells. To further investigate the effects of TGF-β inhibition on OB differentiation in the context of the MM bone marrow microenvironment, we looked at the effect of TGF-β inhibition on the formation of mineralized nodules in the presence of bone marrow plasma collected from three MM patients with extensive bone destruction. Bone marrow plasma from all these MM patients suppressed the development of mineralized nodules (Figure 3B). Treatment with SB431542 restored and enhanced the mineralization suppressed by the bone marrow plasma from MM patients. These results suggest that the inhibition of TGF-β restores OB differentiation and bone formation suppressed in MM.

Because TGF-β inhibition antagonizes the suppressive effects of MM cell conditioned media on OB differentiation (Figure 3A), we next examined whether TGF-β inhibition influences canonical Wnt signaling suppressed by MM cells, using luciferase reporter assays of the T-cell factor (TCF)/ lymphoid enhancer factor (LEF) transcription factor, a downstream target of a canonical Wnt signaling pathway. Addition of MM cell conditioned media suppressed the TCF/LEF reporter activity in MC3T3-E1 cells (Figure 3C). The TCF/LEF reporter activity remained suppressed after the addition of SB431542 at doses high enough to restore OB differentiation, suggesting that TGF-β inhibition does not affect canonical Wnt signaling down-regulated by MM cells.

To further investigate mechanisms of stimulation of mineralized nodule formation by TGF-β inhibition, we next looked at the effect of SB431542 on BMP-2 signaling. Binding of BMP-2 to its cognate receptors enhances phosphorylation of Smad1, a downstream effecter of a canonical BMP-2 signaling pathway. Indeed, addition of BMP-2 induced phosphorylation of Smad1 (Figure 3D). Interestingly, pretreatment with SB431542 further potentiated the phosphorylation of Smad1 by BMP-2. Pretreatment with TGF-β inhibited the phosphorylation of Smad1 by BMP-2; however, addition of SB431542 together with TGF-β restored the BMP-2-induced phosphorylation of Smad1. SB431542 abolished the phosphorylation of Smad2 by TGF-β in MC3T3-E1 cells (Figure 3D), which confirmed the specific inhibition of TGF-beta signaling by SB431542. These results are consistent with the notion that TGF-β inhibition potentiates BMP-2 action and releases OB precursor cells from the blockade of BMP-2 signaling by TGF-β without affecting canonical Wnt signaling down-regulated by MM cells.

### Terminally differentiated OBs suppress MM cell growth and survival

**Figure 4:**
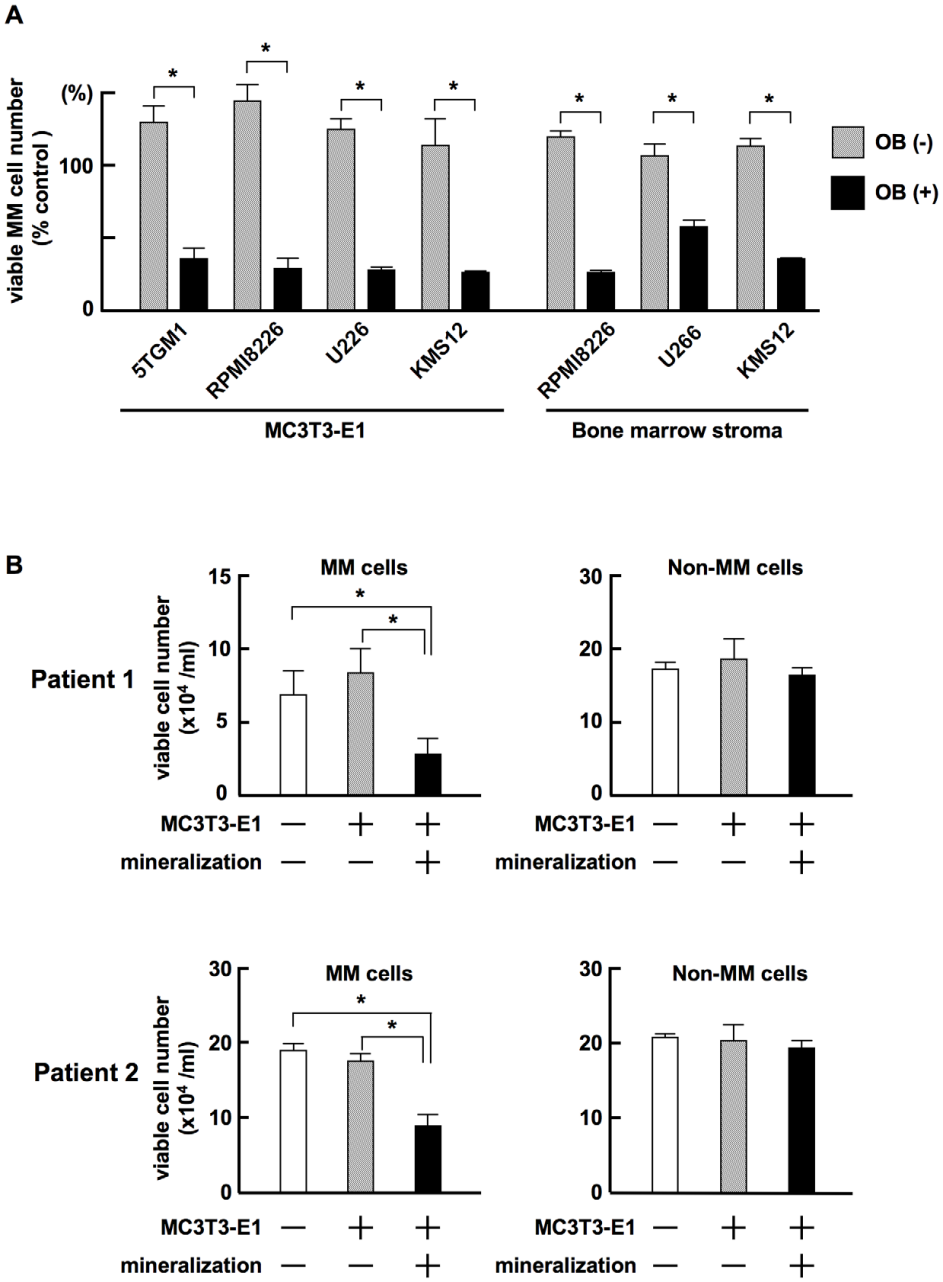
Terminally differentiated OBs suppress MM cell growth. **A.** MC3T3-E1 cells and primary bone marrow stromal cells isolated from a patient with MM were cultured in 24-well culture plates in α-MEM containing 10% FBS with or without β-glycerophosphate, ascorbic acid and 50 ng/mL rhBMP-2 as described in “Materials and methods”. After forming mineralized nodules in the osteogenic cultures with rhBMP-2, the cells were washed to remove rhBMP-2. MM cell lines, 5TGM1, RPMI8226, U266 and KMS-12 at 5×10^4^/mL, were cultured alone as a control or cocultured in quadruplicate with thus treated MC3T3-E1 cells or primary bone marrow stromal cells with or without inducing terminal OB differentiation. After culturing for 3 days, MM cells were harvested, and viable MM cell numbers were counted. Percent changes from the control are shown. Results are expressed as means +/− SD. *, <0.05. **B.** CD138-positive MM cells and CD138-negative non-MM bone marrow cells were immunomagnetically isolated from bone marrow aspirates of MM patients. The cells were cultured alone or co-cultured in triplicate with MC3T3-E1 cells with or without mineralization. After culturing for 3 days, the MM and non-MM bone marrow cells were harvested and viable cell numbers were counted. Data were expressed as means +/− SD. *, p<0.05.

To determine whether induction of OB differentiation in stromal cells affects the MM cell growth-promoting activity of stromal cells, we examined MM cell growth and survival in cocultures with MC3T3-E1 cells or bone marrow stromal cells isolated from a patient with MM with or without induction of terminal OB differentiation. Terminally differentiated OBs forming mineralized nodules derived from MC3T3-E1 cells or primary stromal cells markedly reduced the viability of MM cell lines, while untreated MC3T3-E1 cells or primary stromal cells without OB differentiation did not (Figure 4A). We next determined whether or not the suppression of growth and survival by terminally differentiated OBs is specific to MM cells. CD138-positive MM cells and CD138-negative non-MM cells were immunomagnetically isolated from bone marrow aspirates from MM patients, and cocultured with untreated or terminally differentiated MC3T3-E1 cells. The viability of CD138-positive primary MM cells was substantially decreased after coculturing with MC3T3-E1 cells with mineralized nodules, while CD138-negative normal hematopoietic cells remained intact (Figure 4B). MC3T3-E1 cells without OB differentiation showed suppressive effects on neither CD138-positive nor negative cells. Thus, terminally differentiated OBs suppress the growth and survival of MM cells but not normal hematopoietic cells in the bone marrow.

### Terminally differentiated OBs induce MM cell apoptosis with G_1_ arrest

**Figure 5:**
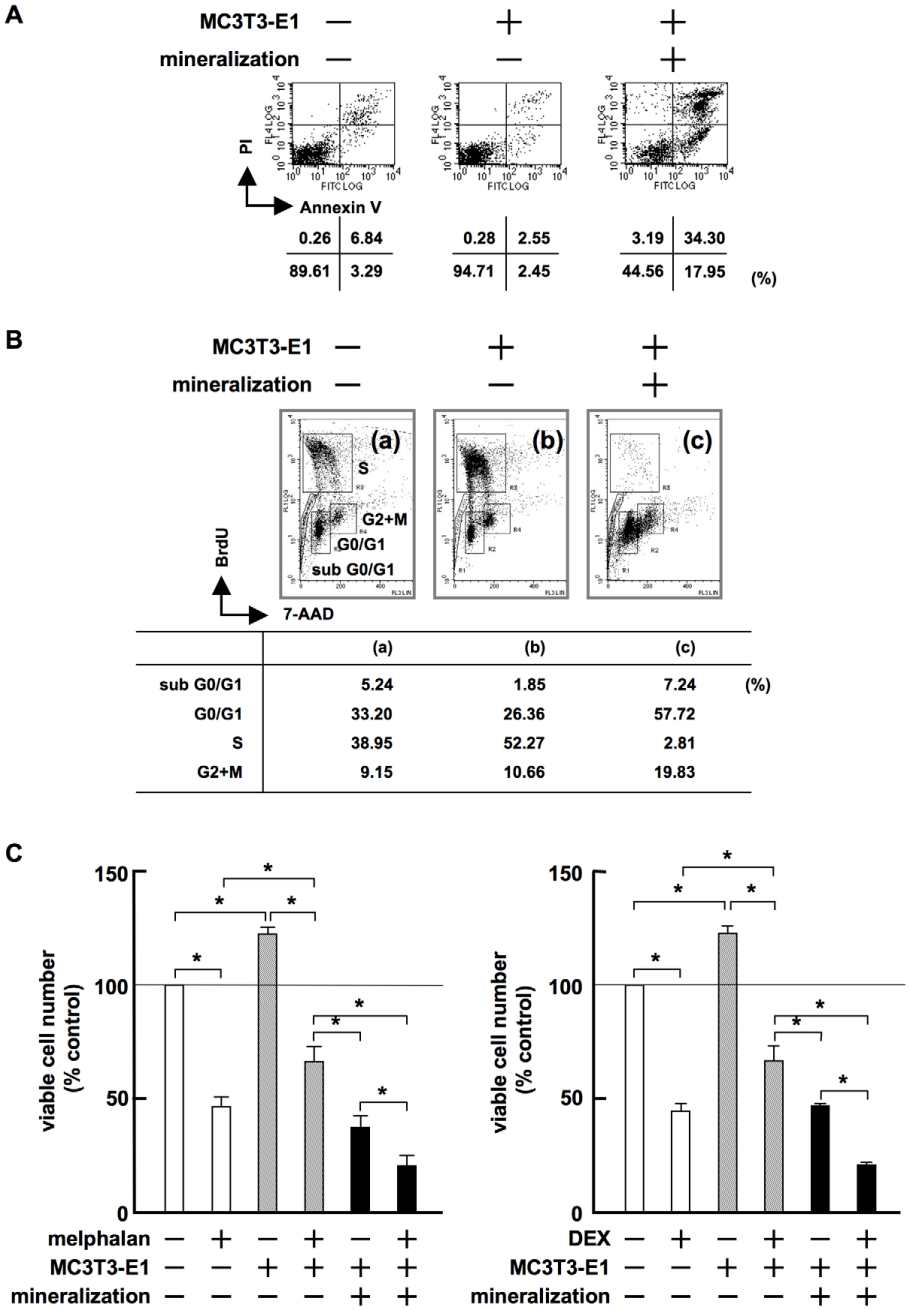
Terminally differentiated OBs induce MM cell apoptosis with G_1_ arrest. **A.** 5TGM1 cells (5×10^4^/mL) were cultured alone or co-cultured with MC3T3-E1 cells with or without mineralized nodules. After culturing for 2 days, 5TGM1 cells were collected, stained with FITC-labeled annexin V and PI, and analyzed by flow cytometry. **B.** 5TGM1 cells were cultured alone, or cocultured with MC3T3-E1 cells with or without mineralized nodules for 2 days. 5TGM1 cells were analyzed by flow cytometry with dual staining of BrdU and 7-AAD as described in “Materials and methods”. Cells were gated corresponding to cell cycle distribution. **C.** RPMI8226 cells were cultured alone or cocultured with MC3T3-E1 cells with or without mineralization in quadruplicate for 3 days in the absence or presence of melphalan at 20 µM (left) or dexamethasone at 10 µM (right). Viable RPMI8226 cells were counted.

We next investigated the effects of terminally differentiated MC3T3-E1 cells on the induction of apoptosis and cell cycle distribution of MM cells. Viable 5TGM1 MM cells slightly increased in number when cocultured with untreated MC3T3-E1 cells (Figure 5A, middle). However, a large number of 5TGM1 MM cells underwent apoptosis with positive staining of annexin V when cocultured with differentiated MC3T3-E1 cells with mineralized nodules (Figure 5A, right). Untreated MC3T3-E1 cells increased the number of 5TGM1 cells in S phase (Figure 5B, middle). In contrast, 5TGM1 cells in S phase almost completely disappeared, and those in G_0_/G_1_ and sub-G_0_/G_1_ substantially increased in number when cocultured with MC3T3-E1 cells with mineralized nodules (Figure 5B, right). These results demonstrate that terminally differentiated OBs induce MM cell apoptosis with G_1_ arrest.

Because stromal cells with suppressed OB differentiation in MM confer drug resistance in MM cells, we next determined whether the induction of terminal OB differentiation can reverse the protective effects of stromal cells on MM cell viability against anti-MM chemotherapeutic agents, melphalan and dexamethasone. Untreated MC3T3-E1 cells attenuated the cytotoxic effects of melphalan (Figure 5C left) and dexamethasone (Figure 5C right) on RPMI8226 MM cells. In contrast, differentiated MC3T3-E1 cells with mineralized nodules did not show protective effects on MM cells and further enhanced the cytotoxicity of these agents against MM cells. These results demonstrate that the induction of terminal OB differentiation can abolish the stromal cell-induced resistance against anti-MM agents, and that the susceptibility of MM cells to anti-MM agents can be reinforced by enhancing the terminal differentiation of OBs.

### Enhancement of OB maturation by TGF-β inhibition facilitates the suppression of MM cell growth

**Figure 6:**
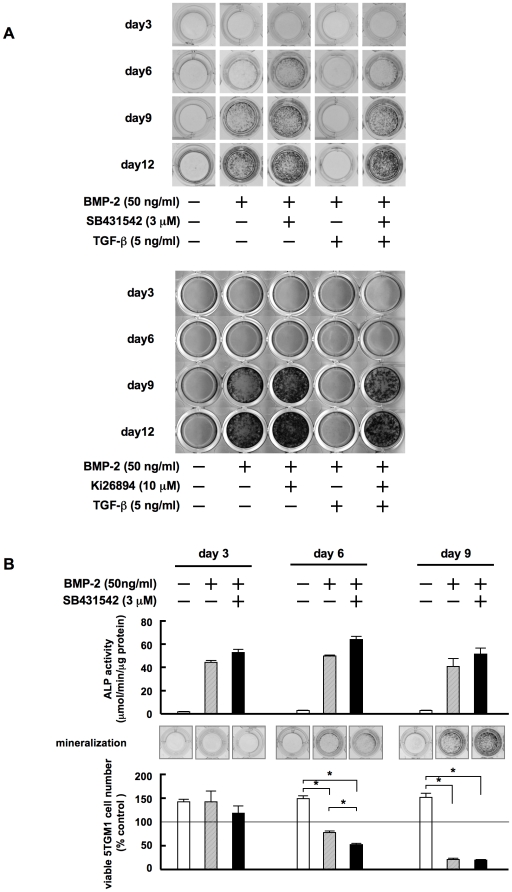
TGF-β inhibition facilitates terminal OB maturation to suppress MM growth and survival at earlier time points. **A.** MC3T3-E1 cells were cultured in osteogenic media for the indicated periods. rhBMP-2, SB431542 and rhTGF-β were added at 50 ng/mL, 3 µM and 5 ng/mL to the indicated wells, respectively. After culturing, mineralized nodules were visualized by von Kossa staining. **B.** MC3T3-E1 cells were cultured in osteogenic media for induction of OB differentiation. rhBMP-2 and SB431542 were added at 50 ng/mL and 3 µM to the indicated wells, respectively. After culturing for 3, 6 and 9 days, the cells were washed, and subjected to the measurement of ALP activity (top panels) and von Kossa staining for assessing mineralized nodule formation (middle panels). The cells were also cocultured in quadruplicate with 5TGM1 cells for 3 days. Viable 5TGM1 cells were counted. Percent changes from the baseline are shown (bottom panels). Data are expressed as means +/− SD. *, p<0.05.

To determine whether TGF-β can expedite OB differentiation, we next sequentially analyzed the effects of inhibiting TGF-β on the formation of mineralized nodules by MC3T3-E1 preosteoblastic cells. SB431542 facilitated OB differentiation as evidenced by the enhanced development of mineralized nodules at day 6, earlier than that with BMP-2 alone (Figure 6A). Addition of TGF-β almost completely suppressed the BMP-2-induced formation of mineralized nodules even at day 12. However, with SB431542 the modulation resumed as early as day 6 even in the presence of TGF-β. These results demonstrate that TGF-β inhibition expedites OB maturation even in a TGF-β-rich milieu.

We next determined whether enhancement of OB maturation by TGF-β inhibition can suppress MM cell growth at earlier time points. After MC3T3-E1 cells had achieved different stages of OB differentiation through culturing with or without BMP-2 and/or SB431542 for 3, 6 and 9 days, they were washed and subsequently cocultured with 5TGM1 MM cells for 3 days. ALP activity in MC3T3-E1 cells was already enhanced at day 3 in the presence of BMP-2 (Figure 6B, upper panels), while no mineralized nodules were observed (Figure 6B, middle panels). Despite the elevation of ALP activity at day 3, 5TGM1 MM cell growth was not suppressed when the MM cells were cocultured with MC3T3-E1 cells at this stage of differentiation (Figure 6B, lower panels). In contrast, suppression of MM cell growth was observed in parallel with the development of mineralized nodules by cocultured MC3T3-E1 cells after 6 and 9 days in the presence of BMP-2 with or without SB431542, and correlated well with the levels of mineralization. These results suggest that terminally differentiated mature OBs with mineralized nodules have the ability to suppress MM cell growth and survival, and that TGF-β inhibition can expedite the differentiation of OBs to suppress MM cell growth and survival.

### TGF-β inhibition suppresses MM cell growth and formation of bone destructive lesions in MM-bearing SCID-rab mice

**Figure 7:**
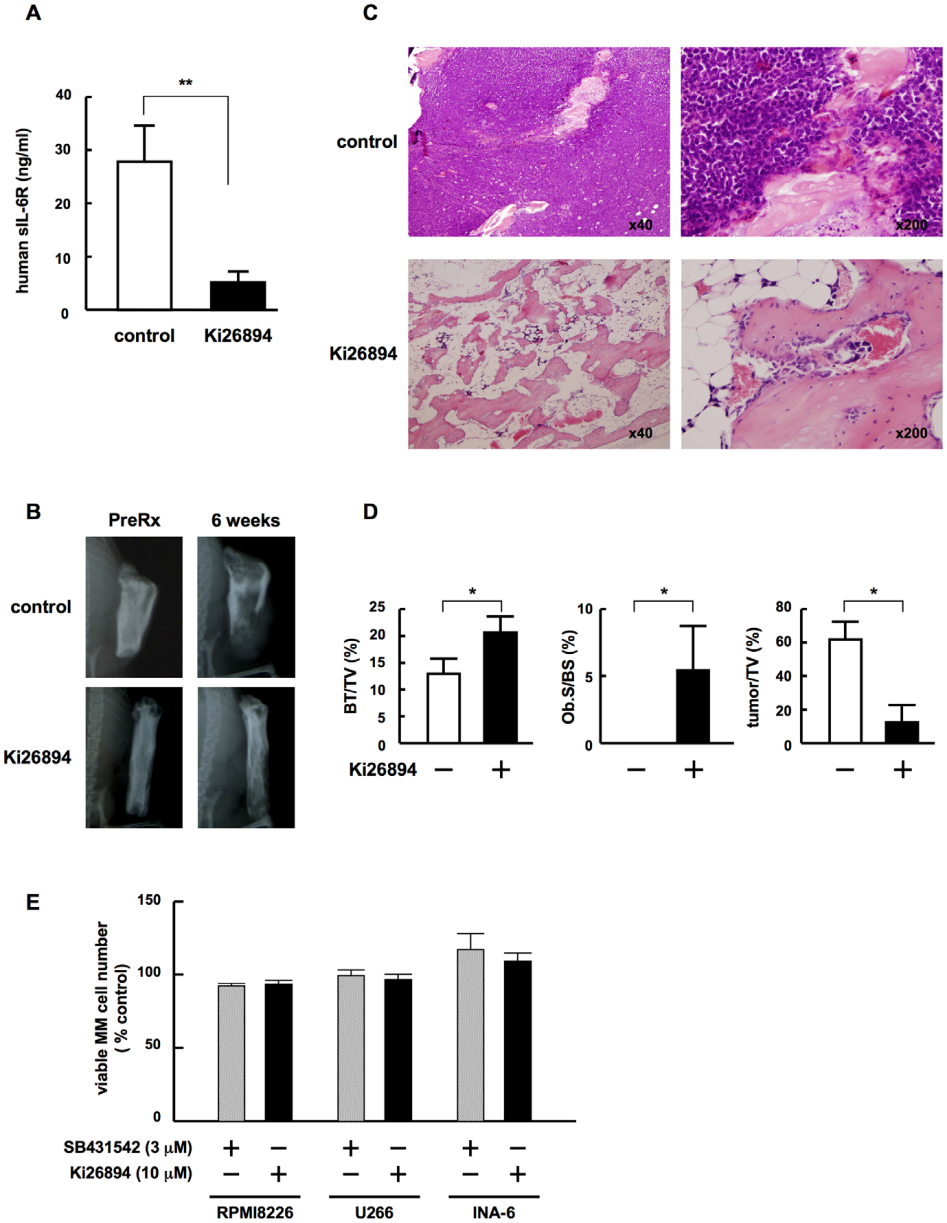
TGF-β inhibition suppresses MM cell growth in bone as well as bone destruction. INA6-bearing SCID-rab mice were given food containing either the TGF-beta type I receptor kinase inhibitor Ki26894 or a vehicle control. **A.** Mouse serum samples were collected 6 weeks after the inoculation of INA6 cells and serum levels of the soluble human IL-6 receptor were measured as described in Materials and methods. **, <0.01. **B.** X-ray photographs of the rabbit bones were taken before and 6 weeks after the treatment. Representative images are shown. **C.** At 6 weeks after the treatment, the rabbit bones were removed. The samples were sectioned and stained with H&E. Representative specimens of control and treated rabbit bones are shown in the upper and lower panels, respectively. Original magnifications were ×40 and ×200 as indicated. **D.** Histomorphometrical analyses of the implanted rabbit bone sections. BV/ TV, Ob.S/BS and tumor/TV were measured in decalcified implanted rabbit bone sections stained with H&E as described in Materials and Methods. Data are expressed as means +/− SD. *, p<0.05. **E.** MM cell lines, INA6, RPMI8226, U266 and KMS-12 at 5×10^4^/mL, were cultured in the absence or presence of Ki26894 (10 µM) or SB431542 (3 µM). After culturing for 3 days, viable MM cell numbers were counted. Percent changes from the control are shown. Results are expressed as means +/− SD.

To evaluate in vivo the effects of TGF-β inhibition on both MM cell growth and the formation of bone destructive lesions, we first established MM-bearing animal models developing a bone disease. Because most MM cell lines grow rapidly and disseminate extraosseously in SCID mice, we utilized an IL-6 or stromal cell-dependent human MM cell line, INA6, which has been shown to hardly grow subcutaneously but grow within a human fetal bone implanted in SCID mice (SCID-hu mice).[27] SCID-rab mice have been developed to substitute for SCID-hu mice to recapitulate MM expansion within the bone marrow and formation of a bone disease.[28] Therefore, we generated SCID-rab MM models using INA6 cells. INA6 cells were inoculated directly into the bone marrow cavity in rabbit bones implanted subcutaneously in SCID mice. INA6 cell-derived human soluble IL-6 receptor was detected as a marker for MM tumor growth 4 weeks after the inoculation of INA6 cells in all the SCID-rab mice, which demonstrates that the rabbit bone microenvironment allows the growth of INA6 cells. Because sera from mice orally treated with the TGF-β type I receptor kinase inhibitor Ki26894 effectively inhibited most of TGF-beta-induced reporter activity,[29] we treated INA6-bearing SCID-rab mice with an oral administration of Ki26894 according to procedures described before.[29] We analyzed MM cell growth and the formation of bone lesions in vehicle- or Ki26894-treated mice 6 weeks after the inoculation of INA6 cells. In vehicle-treated mice, the levels of human soluble IL-6 receptor,[27] a marker for human myeloma tumor burden, in mouse sera were substantially increased (Figure 7A); radiolucent osteolytic lesions were observed in the implanted rabbit bones (Figure 7B). In histological analyses, MM cells were tightly packed in the bone marrow cavity of the rabbit bones while bone trabeculae decreased in size (Figure 7C). However, in Ki26894-treated mice, serum levels of soluble IL-6 receptor remained low (Figure 7A) and only marginal bone destruction was observed on X-ray radiography (Figure 7B). The MM cells markedly decreased in number and formed only small foci in rabbit bones, while bone trabeculae remained without apparent loss of size and structure, and cuboid cells lined the surface of bone, namely osteoblasts (Figure 7C). For quantitative histomorphometric analyses, we measured bone volume per tissue volume (BV/TV) and osteoblast surface per bone surface (Ob.S/BS) of rabbit bones in MM-bearing SCID-rab mice as the indices of bone destruction and osteoblast function, respectively. Both BV/TV and Ob.S/BS were markedly suppressed in vehicle-treated mice. However, both indices retained significantly higher in Ki26894-treated mice, suggesting the protection of bone in vivo by TGF-β inhibition. We also measured tumor per tissue volume (tumor/TV) to assess a tumor burden of the rabbit bones in MM-bearing SCID-rab mice. Tumor/TV was markedly reduced in Ki26894-treated mice (Figure 7D), which is consistent with the serum levels of soluble human IL-6R, a surrogate marker for a human MM tumor burden, in MM-bearing SCID-rab mice. These results suggested that TGF-β inhibition can suppress MM cell growth within the bone marrow while preventing bone destruction and loss.

## Discussion

BMP[30], [31] and canonical Wnt pathways[32], [33] are among the predominant signaling pathways stimulating OB differentiation. A canonical Wnt pathway in OBs is suppressed by soluble Wnt antagonists elaborated by MM cells,[5], [6], [7] stromal cells and OBs.[8], [9] The present study demonstrated that TGF-β plays an important role in arresting the differentiation of stromal cells into mature OBs and that TGF-β suppresses BMP-2 signaling (Figure 3D). In osteolytic lesions in MM which enhance the release and activation of TGF-β, a BMP as well as canonical Wnt signaling pathway in stromal cells and OBs appears to be suppressed, causing severe suppression of OB differentiation. Interestingly, a blockade of TGF-β antagonized the suppressive effects of MM cell conditioned media (Figure 3A) and bone marrow plasma from MM patients (Figure 3B), and was able to release stromal cells from differentiation arrest to achieve terminal OB differentiation. Although the mechanism whereby TGF-β inhibits OB differentiation still remains unclear, we found at least in our experimental conditions that TGF-β inhibition markedly enhances the phosphorylation of Smad1 to potentiate BMP-2 signaling without affecting the canonical Wnt pathway in OB precursor cells suppressed by MM cells (Figures 3C-D). Therefore, the potentiation of BMP-2 signaling at least in part contributes to the restoration of OB differentiation caused by the inhibition of TGF-β.

In the present study, TGF-β inhibition was shown to facilitate terminal OB differentiation in parallel with suppression of MM cell growth and survival. A reverse correlation between OB differentiation and MM tumor growth has recently been reported in patients with MM treated with the proteasome inhibitor bortezomib. Serum levels of bone-specific ALP were found to be elevated after treatment with bortezomib, which were inversely correlated with a reduction in tumor burden.[34], [35], [36] Such bone anabolic effects of bortezomib and their correlation with tumor regression were further demonstrated in MM animal models.[37] MM growth inhibition associated with OB differentiation was also observed in MM animal models treated with other anabolic agents such as anti-DKK1 antibody[38] and lithium chloride[39] as well as with enforced expression of Wnt3a within bone.[40] Together with our results with TGF-β inhibitors, these observations suggest that anti-MM activity emerges with OB differentiation, and that MM cells may protect themselves from such OB-mediated growth suppression by inhibiting the terminal differentiation of OBs.

We found that treatment with the TGF-β type I receptor kinase inhibitor Ki26894 in MM-bearing SCID-rab mice suppressed MM cell growth within the bone marrow while preventing bone destruction and loss (Figure 7). In vivo effects of TGF-β inhibition have also been studied with the TGF-β type I receptor kinase inhibitor SD-208, and shown to increase bone mass in mammary tumor-bearing mice (Mohammad KS, Stebbins E, Kingsley L, et al. J Bone Miner Metab. 2008;23 abstract F275) as well as normal mice.[41] Pharmacological blockade of TGF-β action by the anti-TGF-β monoclonal antibody 1D11 has also been demonstrated to reduce serum M-protein levels as well as improve bone volume and strength in 5TGM1-bearing mouse MM models (Hart AJ, Fowler JA, Lwin ST, et al. J Bone Miner Metab. 2008;23 abstract F293). Together with these in vivo results, our study demonstrates that TGF-β appears to be an important therapeutic target in MM bone lesions. However, because TGF-β inhibitors including SB431542 and Ki26894 did not show direct cytotoxic effects on MM cells (Figure 7E), the combination of TGF-β inhibitors with cytoreductive chemotherapeutic agents or bortezomib may further improve the therapeutic efficacy against MM.

Stromal cells together with OCs create a MM niche in the bone marrow to promote MM cell growth and protect MM cells from spontaneous and drug-induced apoptosis. Stromal cells confer potent drug resistance to blunt the efficacy of anti-MM agents.[14], [15], [16] Importantly, terminally differentiated OBs potentiate cytotoxic effects of melphalan and dexamethasone (Figure 5C), suggesting that mature OBs can increase the susceptibility of MM cells to anti-MM agents to overcome the drug resistance mediated by stromal cells. These results are consistent with a hypothesis that induction of OB differentiation can not only ameliorate destructive bone lesions, but also disrupt the MM niche to suppress MM growth. Furthermore, TGF-β is a multi-functional cytokine which suppresses normal hematopoiesis[42] and dendritic cell differentiation[43] but enhances angiogenesis[44] as well as osteoclastogenesis.[45] Therefore, the effect of TGF-β inhibition may extend beyond amelioration of destructive bone lesions and tumor growth and improve other MM-associated clinical features.

It is intriguing that OBs which have matured enough to form mineralized nodules suppress the proliferation of MM cells in sharp contrast to their precursor, stromal cells, which support MM cell growth and survival. The production of IL-6, a major stromal cell-derived growth and anti-apoptotic factor for MM cells, was found to be markedly decreased in terminally differentiated OBs (91.2±5.7 and 15.1±2.7 pg/mL for 2 days in culture supernatants of untreated and terminally differentiated MC3T3-E1 cells, respectively). Recently, decorin has been identified among OB-derived factors responsible for the suppression of MM cell growth and survival. [46]Profiles of protein production during OB differentiation by a proteome analysis may help identify the OB signature responsible for MM growth suppression.

## Materials and Methods

### Ethics Statement

All procedures involving human specimens were performed under written informed consent according to the Declaration of Helsinki and the protocol approved by the Institutional Review Board for human protection in University of Tokushima (#240). All experiments with animals were performed according to the guidelines for animal protection in University of Tokushima, and approved by the Institutional Review Board for animal protection.

### Reagents

TGF-β type I receptor kinase activin-like kinase 5 (ALK5) inhibitors, SB431542 and Ki26894, were obtained as follows. SB431542 was purchased from Tocris Bioscience (Ellisville, MO). Its IC_50_ for ALK5 is 94 nM.[47] Ki26894 was a gift from Kyowa Hakko Kirin Co. Ltd. (Japan), and is suitable for experiments in vivo.[29] The following reagents were purchased from the indicated manufacturers: recombinant human (rh) bone morphogenetic protein (BMP)-2 and rhTGF-β from R&D Systems (Minneapolis, MN); β-glycerophosphate, melphalan, dexamethasone, rh biglycan, rh decorin and rabbit anti-β-actin polyclonal antibody from Sigma (St. Louis, MO); ascorbic acid from Wako Pure Chemical (Osaka, Japan); rh soluble RANK ligand from Pepro Tech (Rocky Hill, NJ); rabbit anti-Smad1 and rabbit anti-Smad2 from Millipore (Billerica, MA); and anti-phosphorylated Smad1, anti-phosphorylated Smad2 and horseradish peroxidase–conjugated anti-mouse IgG from Cell Signaling (Beverly, MA).

### Cells and cultures

Human MM cell lines, RPMI8226 and U266, were obtained from the American Type Culture Collection (Rockville, MD). The human MM cell line KMS-12 was obtained from Health Science Research Resources Bank (Osaka, Japan). The MM cell line INA6 was kindly provided by Dr. Renate Burger (University of Kiel, Kiel, Germany). The mouse MM cell line 5TGM1 was a gift from Dr. Gregory R. Mundy (Vanderbilt Center for Bone Biology, Vanderbilt University, Nashville, TN). A mouse preosteoblastic cell line, MC3T3-E1, and an immature mesenchymal cell line, C3H10T1/2 were obtained from RIKEN Bioresource Center (Tsukuba, Japan). Human bone marrow mononuclear cells were isolated by Ficoll-Hypaque density gradient centrifusion from heparinized bone marrow blood drawn from patients with MM. MM cells were further purified from bone marrow mononuclear cells with positive selection using CD138 (Syndecan-1) microbeads and the Miltenyi magnetic cell-sorting system (Miltenyi Biotec, Auburn, CA) according to instructions. Primary stromal cells were isolated from bone marrow aspirates and cultured as previously described.[5] Cells were cultured in Minimal Essential Medium Eagle-Alpha Modification (α-MEM; Sigma) with 10% heat-inactivated fetal bovine serum (FBS; SAFC Biosciences, Lenexa, KS) or 1% FBS as a serum-reduced condition, 100 units/mL penicillin(Sigma) and 100 µg/mL streptomycin (Sigma). Conditioned media were collected from MM cell lines cultured at 5×10^5^ cells/mL for 2 days. Bone marrow plasma was obtained after the centrifusion of heparinized bone marrow blood drawn from patients with MM.

### OB differentiation

MC3T3-E1 pre-osteoblastic cells and bone marrow-derived stromal cells were cultured in 24-well culture plates in an osteogenic medium, α-MEM containing 10% FBS, 10 mM β-glycerophosphate and 50 µg/mL ascorbic acid, as previously described.[5] The medium was replaced every 3 days. Alkaline phosphatase (ALP) activity was determined using an ALP activity assay kit (Wako Pure Chemical) according to the manufacturer's instructions. For analyzing mineralized nodules, the cells were fixed with 10% neutral-buffered formalin (Wako Pure Chemical) and visualized by von Kossa staining as described.[5]

### Adipogenic differentation

C3H10T1/2 cells were cultured in an osteogenic medium with BMP-2. On day 7 of adipogenic induction, cells were washed, fixed and stained for intracellular lipid inclusion bodies with 0.5% oil red O (Sigma) in isopropanol:distilled water (60∶40) for 30 minutes.

### RT-PCR

Total RNA was extracted from cells using TRIZOL reagent (Gibco BRL, Rockville, MD). For reverse transcription-polymerase chain reaction (RT-PCR), 2 µg of total RNA was reverse-transcribed with Superscript II (Gibco) in a 20-µl reaction solution. One tenth of the RT-PCR product was used for subsequent PCR analysis with 24–30 cycles of 95°C for 30 seconds, 58°C for 30 seconds, and 72°C for 30 seconds.

### Transfection

MC3T3-E1 cells were seeded in 6-well culture plates at 50% confluence and co-transfected with 2 µg of chimeric firefly luciferase reporter plasmid with Renilla luciferase reporter plasmid (pRL-TK; Promega, Madison, WI) as an internal control using GenePorter2 transfection reagent (Genlantis Inc., San Diego, CA) in opti-MEM (Invitrogen, Carlsbad, CA) supplemented with 1% FBS. A TCF/LEF binding site reporter plasmid was used (Topflash™; Upstate Biotechnology, Lake Placid, NY). The medium was replaced with αMEM containing 1% FBS at 16 hours after transfection. For dual-luciferase assays, the cells were washed and lysed with a passive cell lysis buffer (Promega, Madison, WI), and both firefly and renilla luciferase activity was measured with a luminometer (ATTO, Tokyo, Japan) by mixing the luciferase substrate (Promega) with 10 µL of cell lysate. Transcriptional activity was expressed as renilla luciferase activity.

### Western blot analysis

Cells were collected and lysed in a lysis buffer (Cell Signaling) supplemented with 1 mM phenylmethylsulfonyl fluoride and protease inhibitor cocktail solution (Sigma). Cell lysates were separated by SDS-PAGE and transferred to polyvinylidene difluoride membranes (Millipore). Membranes were blocked with 5% non-fat dry milk in TBS with 0.01% Tween 20 (MP Biomedicals, Irvine, CA) for one hour at room temperature and incubated for 16 hours at 4°C with anti-phospholylated-Smad1, anti-Smad1, or anti-β-actin antibody. After washing, secondary horseradish peroxidase–conjugated antibody was added, and the membranes were developed using the Enhanced Chemiluminescence Plus Western Blotting Detection System (Amersham Biosciences, Piscataway, NJ).

### Cell viability and cell cycle analysis

Viable cell numbers were measured by a cell proliferation assay using 2-(2-methoxy-4-nitrophenyl)-3-(4-nitrophenyl)-5-(2,4-disulfophenyl)-2H-tetrazolium (WST-8; Kishida Chemical, Osaka, Japan), or a trypan blue exclusion assay, as previously described.[12] Apoptosis was evaluated by staining cells with an annexin V-FITC and propidium iodide (PI) labeling kit (MEBCYTO Apoptosis Kit; MBL,Nagano, Japan) according to instructions. Cell cycle distribution was evaluated by staining cells with a bromodeoxyuridine (BrdU) and 7-amino-actinomycin D (7-AAD) labeling kit (BrdU Flow Kit; BD Pharmingen, San Diego, CA) according to instructions. Briefly, cells were cultured with BrdU at 10 µM for the last 2 hours of culture. They were collected, fixed, and incubated with a FITC-conjugated monoclonal antibody against BrdU. 7-AAD (20 µL per sample) was added for DNA staining. BrdU incorporation and DNA content were analyzed by flow cytometry.

### MM mouse model and measurement of serum soluble human IL-6 receptor levels

The SCID-rab MM mouse model was prepared as described.[28] Briefly, femora and tibiae from 4-week-old Japanese white rabbits (Kitayama Labes, Nagano, Japan) were implanted subcutaneously into six-week-old male CB-17 SCID mice (CLEA Japan, Tokyo, Japan). After allowing bone engraftment for 4 weeks following the implantation, 1×10^6^ INA-6 cells in 50 µL of phosphate-buffered saline (PBS) was inoculated directly into the bone marrow cavity of the rabbit bones. A TGF-beta type I receptor kinase inhibitor, Ki26894, was mixed with food (0.08%) and administered orally via chow as previously reported.[29] The treatment with Ki26894 was initiated 1 day after the inoculation of INA6 cells. Serum levels of soluble human IL-6 receptor derived from INA6 cells were used as a marker for tumor burden as previously described.[27] Mouse sera were serially collected and serum levels of soluble human IL-6 receptor were measured with an enzyme-linked immunosorbent assay (human IL-6 sR; R&D Systems, Minneapolis, MN). Six weeks after the MM cell inoculation, the mice were anesthetized with an i.p. injection of pentobarbital (Dainippon Pharma, Osaka, Japan). X-ray photographs of the rabbit bones were taken, and the rabbit bones were collected. The rabbit bones were fixed in 10% phosphate-buffered formalin, and decalcified with 10% EDTA. The samples were further embedded in paraffin, and sectioned. Sections were stained with hematoxylin and eosin (H&E) for histopathologic examination. Bone volume per tissue volume (BV/TV), osteoblast surface per bone surface (Ob.S/BS) and tumor per tissue volume (tumor/TV) were measured in decalcified implanted rabbit bone sections stained with H&E as the indices of bone destruction, osteoblast function and a tumor burden, respectively, as described previously. [48], [49]

### Quantification of data

Raw image data of mineralized nodule formation and immunobolotting were quantified with the Multi Gauge version 3.0 software (Fujifilm, Tokyo, Japan).

### Statistical analysis

Statistical significance was determined by a one-way analysis of variance (ANOVA) with Scheffe post hoc tests. The minimal level of significance was a p value equal to 0.05.
